# Postgenomic Bioinformatic Analysis of Nucleic Acid Sequences Expressed in the Salivary Gland Epithelial Cells of Primary Sjögren’s Syndrome Patients in Search of Microorganisms and Endogenous Retroviruses

**DOI:** 10.31138/mjr.33.3.371

**Published:** 2022-09-30

**Authors:** Menelaos N. Manoussakis, Ioanna P. Svolaki, Orsalia Hazapis, Loukia Zerva, Vassilis G. Gorgoulis, Gkikas Magiorkinis

**Affiliations:** 1 Laboratory of Cellular and Molecular Immunology, Department of Pathophysiology, School of Medicine, National and Kapodistrian University of Athens, Athens, Greece,; 2 Molecular Carcinogenesis Group, Department of Histology and Embryology, School of Medicine, National and Kapodistrian University of Athens, Athens, Greece,; 3 Department of Hygiene Epidemiology and Medical Statistics, School of Medicine, National and Kapodistrian University of Athens, Athens, Greece

**Keywords:** Sjögren’s syndrome, epithelial cells, sequencing, infections, retroviruses

## Abstract

Several previous studies from our laboratory have indicated that the salivary gland epithelia of primary Sjögren’s syndrome (SS) patients are not only the target of autoimmune immune responses, but also key instigators of the chronic salivary gland inflammatory infiltrates of patients. In particular, the comparative analysis of salivary gland tissue specimens and of in-vitro cultured non-neoplastic salivary gland epithelial cell lines (SGEC, of ductal type) from SS-patients and non-SS disease-controls, have unequivocally highlighted the presence of intrinsic activation in the ductal epithelia of SS-patients and of aberrant expression of inflammagenic molecules thereof, that correlate with the severity of local histopathologic changes, as well as of systemic manifestations of the disease. In the same context, we have recently shown that the ductal epithelia of SS-patients manifest cell-autonomous activation of the AIM2 inflammasome owing to the presence of aberrant cytoplasmic accumulations of damaged DNA. These findings not only provide a mechanistic explanation for the intrinsic activation and inflammatory status of SS ductal epithelia, but may also point towards the putative instigating role of an exogenous or endogenous agent (i.e., a micro-organism or an endogenous retrovirus, respectively). On this basis and to further explore the nature of epithelial cell-intrinsic activation in SS, the present proposal aims to investigate the expression of endogenous retroviral and/or non-human nucleic acid sequences of microbial origin in the ductal salivary gland epithelia of SS-patients, using metagenomic analysis of high throughput DNA and RNA genome sequencing data, which will be obtained from SGEC lines derived from SS-patients and disease-controls.

## THEORETICAL BACKGROUND

Primary Sjögren’s syndrome (SS) or autoimmune epithelitis is a chronic autoimmune rheumatic disease mainly characterized by dysfunction and destruction of the exocrine glands (particularly of the salivary and lacrimal glands) and the presence of chronic peri-ductal lymphocytic infiltrates in the affected glands. SS may extend from disease confined to the exocrine glands (organ-specific exocrinopathy) to the expression of various extraglandular manifestations (systemic disease) and the development of B-lymphocyte lymphoma (non-Hodgkin’s lymphoma, usually MALT). Although the pathogenesis of the disease remains largely unclear, several lines of evidence presented over the last 25 years from our group and others indicate the important pathogenetic role of the ductal salivary epithelia, acting as instigators of chronic inflammatory reactions in the salivary gland (SG) tissues of patients.^1,2^ Toward these studies, we have developed a reproducible method for the establishment of long-term cultured non-neoplastic salivary gland epithelial cell (SGEC) lines from minor salivary gland biopsies, which express the ductal epithelial phenotype and are devoid of other types of cells and the influence of tissue microenvironmental factors thereof.^3^

Multiple comparative studies of SGEC lines derived from patients with SS (SS-SGEC lines) and from disease controls (control-SGEC lines derived from patients with sialadenitis, who did not fulfil the classification criteria for SS) have indicated that SS-SGEC lines manifest an intrinsic (cell-autonomous) activation phenotype and various functional aberrations thereof, which closely correlate to the histological and systemic manifestations of the disease.^2^ Among others, the transcriptome analyses of SS-SGEC lines have revealed constitutive perturbations in several inflammation- and metabolism-related signalling pathways, which were more pronounced in cell lines derived from patients with severe histopathologic lesions.^4^ In addition, we have recently shown that the SS-SGEC lines exhibit cell-autonomous activation of the AIM2 inflammasome, as indicated by the intrinsic activation of the AIM2 molecule and caspase-1, the formation of ASC-specific complexes (ASC specks) and the spontaneous production of high IL-1β protein levels.^5,6^ AIM2 activation was also confirmed in-situ in the salivary ductal epithelia of SS patients with severe inflammatory lympho-epithelial infiltrations.^6^ Furthermore, this study revealed significant cytoplasmic accumulations of damaged DNA, both in-vitro in the SS-SGEC lines and in-situ in patients’ epithelia, a finding that provides a mechanistic explanation for the cell-autonomous AIM2 activation and all together for the intrinsic activation and inflammagenic properties of the ductal epithelium of SS patients.^6^

It is well established that both genetic and environmental factors are involved in the induction and maintenance of autoimmune diseases, such as SS. Probably the most important indication for the pathogenetic involvement of a non-genetic factor is provided by the relatively small correlation for the development of autoimmune disease in monozygotic twins (less than 50%).^7,8^ Although the list of possible environmental (non-genetic) factors that may precede the development of autoimmune disease is extensive, a number of data suggest that an infection, as a causative agent of SS, remains a strong possibility. In particular, some infections have been shown to cause SS-like diseases,^9^ and, as a result, it has been suggested that they may be directly involved in the pathogenesis and induction of SS, while others may provide a protective role.^10,11^ Infectious agents that mimic SS include tuberculosis, leprosy, malaria, subacute bacterial endocarditis and spirochete infections, hepatitis A, B and C viruses, parvovirus B19, and HIV. In addition, certain viruses express tropism for salivary and lacrimal glandular tissues, and are particularly related to the herpesvirus family including CMV, EBV, HHV-6, HHV-7 and HHV-8.^9^ Finally, a number of data have suggested, albeit inconclusively, the possible role of endogenous retroviruses,^12–14^ hepatitis D virus,^15^ as well as retroviruses (HTLV-1, HIV, HIAP-I and HRV-5 SS)^16^ in the pathogenesis of SS.

So far, the unfulfilled search for an infectious agent with putative pathogenetic role in SS may largely owe to the absence of thorough studies of the ductal epithelium of patients, which represents the predominantly affected tissue in this disorder. In fact, as mentioned above, the ductal epithelia of SS patients manifest major signs of chronic cellular stress, which may be an indication of incitement by an exogenous infectious agent (microorganism) or of endogenous viral elements in the genome (endogenous retroviruses). Over the last decade, the emergence of high-throughput sequencing has revolutionized the field of genomic medicine. Among other, the postgenomic analysis of the complete human tissue genome has led to the identification of DNA/RNA of microbial origin (i.e., from bacteria, fungi, protozoa or viruses) in a variety of clinical specimens.^17–20^

## METHODS

All samples to be studied have been collected or will be collected after informed written consent by patients and controls. The study has been approved by the Bioethics Committee of the School of Medicine, National University of Athens (protocol no. 5107). The following methodologies will be used in this research proposal:

### Development of cultured non-neoplastic salivary gland epithelial cell lines (SGEC) of ductal type

Labial minor salivary gland (MSG) biopsy specimens will be obtained with informed consent from individuals during their diagnostic evaluation for SS and overall will include 7 from SS patients with systemic manifestations and 3 from non-SS disease controls, who do not fulfil the European-American classification criteria for SS.^21^

MSG samples will be immediately fixed in standard formalin before paraffin embedding and biopsy focus score (number of lymphocytic foci per 4 mm^2^ of tissue area) will be determined as previously.^3^ Long-term cultured non-neoplastic secondary SGEC lines will be established from the MSG biopsies of SS patients and controls (SSSGEC and control-SGEC lines, respectively) and will be maintained in serum-free keratinocyte basal medium (Clonetics), as previously described.^3^ Thus, established SGEC lines express the ductal epithelial phenotype and are devoid of other types of cells and the influence of tissue microenvironmental factors thereof.^3^ The exclusive epithelial nature and ductal epithelial origin of cultured SGEC lines was routinely verified by morphology, as well as by the uniform and consistent expression of epithelial-specific markers and the absence of markers indicative of other types of cells.

### Preparation of nuclear DNA, DNA sequencing, complete genome bioinformatic analysis and postgenomic DNA analysis

In brief, epithelial cells will be collected after trypsinization of SGEC lines (7 from SS patients and 3 from non-SS disease controls) and nuclear DNA will be isolated with the PureLink™ Genomic DNA Purification Kit (Life Technologies). The isolated nuclear DNA will be subjected to high-throughput sequencing (Illumina paired-end), to a target average coverage depth of 30x and a read length of 150 bp.^22^ Variant calling analysis will be performed following alignment with the human reference genome (hg38 version). Briefly, the Bowtie2 program (https://pubmed.ncbi.nlm.nih.gov/22388286/) will be used, as well as the view command of the Samtools with the -q option to ensure sequence quality higher than 20 (https://pubmed.ncbi.nlm.nih.gov/19505943/). The IGV (Integrative Genomics Viewer) tool will be used to visualize the mapped arrays (https://pubmed.ncbi.nlm.nih.gov/21221095/). FastQC will also be used to assess read quality (i.e., to ensure low levels of double-reading of sequences included, https://www.bioinformatics.babraham.ac.uk/projects/fastqc/). Sequences not mapped into the human genome will be studied in detail by post-genomic analysis against an existing microbial sequence database, in order to detect and identify the possible existence of non-human microorganisms (https://pubmed.ncbi.nlm.nih.gov/32055707/). Finally, the results will be further tested by PCR and classic sequencing (Sanger sequencing) to confirm the findings.

### Bioinformatic analysis of already available complete RNA sequencing (RNA-seq) results for the detection of retroviruses and RNA viruses

Paired-end total RNA sequencing results will be used, which we have recently obtained from 30 SGEC lines (15 from SS patients and 15 from non-SS disease controls, https://www.precisionmedicine.gr/1st- open-call, pMedGR, Center of New Biotechnologies & Precision Medicine of the Medical School of the National and Kapodistrian University of Athens). The analysis of RNA-seq data will implement algorithms developed and recently published by the Applied Evolutionary Virology group at the Department of Hygiene, Epidemiology and Medical Statistics (https://journals.asm.org/doi/full/10.1128/Spectrum.01260-21). In summary, specialized algorithms take into account the similarity between insertions of endogenous retroviruses, allowing the estimation of their expression levels (per retroviral family), which will subsequently be normalized, according to the expression of house-keeping genes. To detect and identify putative non-human microorganisms, sequences, which will not be mapped into the human genome, will be subjected to extensive post-genomic analysis against an existing microbial sequence database (https://pubmed.ncbi.nlm.nih.gov/32055707/). Finally, the results will be further tested by PCR and classic sequencing (Sanger sequencing) to confirm the findings.

## IMPORTANCE OF THE STUDY

The possible detection of the presence of genome of exogenous origin (microorganism) or endogenous origin (endogenous retroviruses) in the epithelial cells of the salivary glands of SS patients may allow the elucidation of the pathogenetic mechanisms that underlie this disorder. Several lines of evidence indicate that the intrinsic ductal epithelial activation in SS patients contributes to the development of systemic disease and B-cell lymphoma observed. In this context, the proposed study is expected to contribute to the understanding of the pathogenesis of the above disease manifestations.
